# Experiences of health care in family members of persons with incurable oesophageal or gastric cancer – a hermeneutical phenomenological study

**DOI:** 10.1186/s12904-026-02380-x

**Published:** 2026-10-13

**Authors:** Sofia Kårmark, Marlene Malmström, Jimmie Kristensson

**Affiliations:** 1 Department of Health Sciences, Faculty of Medicine, Lund University, Box 117, Lund, 221 00 Sweden; 2 Skåne University Hospital, Lund, Sweden; 3 Institute for Palliative Care, Lund University and Region Skåne, Lund, Sweden

**Keywords:** Family members, Oesophageal cancer, Gastric cancer, Palliative care

## Abstract

**Background:**

Cancer affects patient’s family members, who often become informal caregivers. For those with an incurable illness a palliative care approach is important. The approach includes need and support for patients and family members. Early integrated PC has been proven beneficial for patients with oesophageal or gastric cancer. However, the knowledge about family members’ burden is general and lacks in specificity. It is argued that early PC needs to be diagnosis specific to be successful. Previous research has shown that family members of cancer patients in general experience cancer care as unsatisfying and somewhat problematic. The specific needs, in terms of health care of family members of persons with incurable oesophageal or gastric cancer are unclear. This study therefore aims to capture their lived experiences of health care.

**Method:**

This study was conducted as a qualitative interview study during 2021–2023 and analysed with hermeneutic phenomenology according to the method described by Max van Manen. The analysis was inspired by Van Manen’s analytical steps to find the essence of the phenomenon of health care experiences. Furthermore, in order to identify these, the analysis was guided by Van Manen’s description of lived experiences through the five existentials of the human being: *Corporality, Temporality, Spatiality, Relationality* and *Materiality*.

**Results:**

The findings were captured in the main theme “To strive and struggle”, which described the lived hardship of health care experiences by family members. The strives and struggles were identified in different areas and five sub-themes described these: “*The body as a tool*”, “*Time is out of order*”, “*Navigating in health care spaces*”, “*To be in the hands of the right persons*” and “*To learn and develop new skills*”.

**Conclusion:**

This study adds to the knowledge on health care experiences of family members of those with incurable oesophageal or gastric cancer. It suggests that supporting and guiding family members through the fragmented health care system by introducing integrated early PC may decrease anxiety, increase the likelihood to form trusting relationships and lead to fewer emergency department visits.

**Supplementary Information:**

The online version contains supplementary material available at https://doi.org/10.1186/s12904-026-02380-x.

## Background

Every year approximately 1300 persons are diagnosed with oesophageal or gastric cancer living in Sweden. Just over 50% of them have an illness that is incurable and only 20% live longer than a year after the diagnosis [1–4]. Cancer as an entirety engages more than the ill person. It also has a severe impact on family members, often leaving them troubled by depression symptoms and depressed health and, sometimes, social challenges as well as poor quality of life [5–12]. Family members’ existential, psycho-social and physically supportive role towards the ill person is well recognized. It has been found to be common for family members to be part of the ill person’s cancer trajectory and become informal caregivers [8, 13, 14]. A family caregiver or informal caregiver is someone who is involved in the management of the disease, and who performs caregiving acts without having a formal assignment or getting paid [15–17]. A family member in this context is an individual whom the ill person has chosen and considers a close friend or relative.

Family members’ need for support is an important component of palliative care (PC). The approach aims not only to provide the ill person with care and support throughout the disease course but also to support the family members surrounding them, meeting their physical, psychosocial and existential needs. Palliative care is an approach that strives to decrease patients symptom load and increase their quality of life [18]. International studies indicate that relational aspects are essential to the experiences of good PC, with staff’s medical competence, person-centredness, proactiveness and availability as the most important components [19]. Both the World Health Organization (WHO) and recent research within the field enhance the importance of PC being initiated early in the disease trajectory and being integrated with health care [20–23]. According to the American Society of Clinical Oncology (ASCO), early PC is recommended to be initiated 8 weeks after a diagnosis with PC needs [5]. It has been shown that it is important for effective family support to be included in early palliative cancer care and that family members experience support and guidance [24, 25]. Several studies show that, for an early PC approach to be successful and for patients to benefit, it needs to be tumour specific or tailored to the specific illness with its specific symptoms and needs [23, 26, 27]. This suggest that the same may apply to family members. On the one hand, research suggests that PC should be diagnosis-specific and integrated early; on the other, PC is an approach for support based on needs. This study focuses on diagnosis-specific aspects for family members.

Family members of persons with cancer have various experiences of health care in relation to the needs of and interventions for the ill person and themselves. It has previously been shown that they experience cancer care as fragmented, with a lack of continuity, and report that practical information is insufficient and that routines for family involvement are lacking. Furthermore, experiences of emotional challenges and feelings of invisibility in family members have been linked to health care interactions; and, compared with health care personnel, family members seem to have different perceptions of time in relation to health care interventions for the ill person [19, 28, 29]. To understand the complexity of the individual and mutual cancer management, health care ought to approach persons with cancer and their caregiving family members as a family unit [17, 30].

In oesophageal or gastric cancer, research previously focused on experiences of the family members of those receiving treatment with a curative intent. Perceptions of the family members as having sole responsibility for the ill person’s continued care, and feelings of abandonment after hospital discharge from a surgical curative intervention were reported [31]. For those with incurable oesophageal or gastric cancer, family members are reportedly involved in all aspects of the disease course from diagnosis until end -of -life, while simultaneously experiencing great loneliness [32]. The specific disease trajectory that they are facing is rapid and characterized by a decline in quality of life and severe symptom load. The symptom load largely consists of nutritional issues, which affect family members both socially and in their everyday life. Moreover, family members have to manage the fact that the ill person receiving palliative treatment has a higher frequency of unplanned hospital stays and outpatient care visits compared with those receiving curative treatment [2, 3, 32–36] although early PC, has been related to less acute hospital admissions [37].

Family members of cancer patients in general are highly involved and numerous studies describe their health care experiences regardless of treatment intention, revealing areas for supporting family members [9, 17, 19, 28, 29]. Despite knowledge about the extensive family involvement and feelings of loneliness, little is known about the experiences with cancer care of family members of persons with incurable oesophageal and gastric cancer. This patient group’s nutritional symptom load [2, 3, 32–34], frequent health care interactions [36], family members' great involvement in the management of the disease [32] and unsatisfying experiences in relation to treatable intentions [31], imply that specific illness experiences are of essence in relation to health care and support, also suggested by studies concerning early PC [23–27]. However, there is as yet no research referring to the Swedish health care context for family members of persons who are incurably ill in the oesophagus and stomach. In order to offer family members sufficient support in early PC, a knowledge expansion may therefore be useful.

## Aim

The study’s aim was to capture the lived experiences of health care in family members of persons with incurable oesophageal or gastric cancer.

## Design

This study was conducted using a qualitative inductive hermeneutic phenomenological design [38, 39]. Hermeneutic phenomenology is a method used for investigating a lived human phenomenon such as the experience of health care, which is done by gathering lived experiences. The hermeneutic perspective is characterized by an openness in the researcher, who has to try to understand their own pre-understanding and the effect it may possibly have on the results.

The study was guided by the hermeneutic phenomenological perspectives of Max Van Manen and his perspectives of understanding lived experience. According to Van Manen each human being’s lived experiences can be understood through components known as existentials. An existential is a universal theme of life that humans experience their reality through and together they constitute a humans experience of a life [40]. There are six basic existentials listed by van Manen, they are; the experiences of the lived body- *Corporeality*, the experiences of lived time- *Temporality*, the experiences of lived space- *Spatiality*, the experiences of the lived self in relation to others- *Relationality*, the experiences of lived things- *Materiality*, and with the more recent addendum the experiences of lived cyborg relations- *Experiencing technology*. In this study all of these except *Experiencing technology* contributed to understanding family member’s lived experiences of health care [38, 41, 42].

### Context

Oesophageal and gastric cancer are diagnosed in Sweden’s six national centres assigned to investigating and treating these cancers, in the 21 tax-funded health care regions [43]. Each patient in one of the six centres is discussed during a multidisciplinary conference whose main focus is to agree on a treatment strategy that the patient is presented with, which is then documented. Providing good information to family members is part of the national centre’s role when diagnosing and presenting the treatment strategy. However, there are no guidelines for how to support family members, for instance concerning travel.

### Participants

The study comprised of twelve participants who were recruited at one of the nationally assigned centres for oesophageal and gastric cancer. Aiming to invite family members, patients with a diagnosis of incurable oesophageal or gastric cancer during 2021–2023 were identified retrospectively in the documentation, following the multidisciplinary conference with the help of a nurse. “Incurable” in this context was defined as receiving treatment with a palliative intent treatment regardless of reasons (tumour, node, metastasis (TNM) stage, personal treatment references etc.). The patients received a letter with the question whether they had a family member who might be approached about participating in the study. Consent from the patient-family member pair and the family member’s contact information were returned by letter to the researcher who then contacted the family member to confirm the consent and to set up an interview. Inclusion criteria for the study were consequently family members of patients with a diagnosis of an incurable oesophageal or gastric cancer who were > −18 years old, fluent in Swedish, and had no diagnosed cognitive impairment. It was planned to perform a strategic selection of participants but the low number of consenting family members throughout the recruitment process meant that all identified family members had to be approached about participation.

However, the sample randomly turned out to be varied concerning point of time in the patient’s disease trajectory, ranging from one month after diagnosis to after death (four interviews). An invitation letter was sent to 68 patients in total, 52 men and 16 women. Forty-nine family members did not respond, Five patients declined (all men) and fourteen agreed to participate in the study (14 women). Before interview start one participant withdrew their participation and one was not reachable for setting up the interview. In total twelve interviews were performed. All but one, a mother, interviewed participants were female partners of the patient. Age was recorded for six participants and varied between 64 and 80 years. One participant chose to have the patient present during the interview; the remaining interviews were carried out with only the family member. The recruited patients comprised of 11 men and one woman; two had incurable cancer in the stomach and ten in the oesophagus.

### Data collection

Data were collected through semi-structured interviews consisting of questions aiming to capture lived experiences without the mention of existentials (Supplementary file). The interview started with an open question and was built around a timeline symbolizing the patient’s disease trajectory. The timeline was placed in front of the participant and aimed to give an overview of the trajectory. Each family member was asked before the interview to refer to the timeline, show where the different experiences occurred on it, as well as indicate the starting and finishing point. Although each had a choice, all participants preferred that the interviewee wrote the notes on the timeline during the interview.

The interviews varied between 53 and 113 min, with an average of 79 min and a median of 70.5 min. Ten interviews were performed face to face and two by phone, one because of a considerable geographical distance and one at the request of the participant. After twelve interviews the transcripts were read through, and data were deemed sufficient with no new data emerging.

### Data analysis

The data were analysed inspired by Van Manen and his three steps of analysis. Van Manen’s existentials, with the exception of *Experiencing technology*, guided the analysis [38, 39, 44]. The interview questions were open and not focusing on specific existentials (supplementary file). Focus on the existentials occurred during the analysis, consequently guiding the process (Table 1).

**Table 1:** Description of analytical process

**Text**	**Holistic reading**	**Essence of the text**	**Relating to existential**	**Theme**
“*On the occasions when I have called and needed someone from the home healthcare service to come, they have come and assessed the situation. I think they have been helpful, about 98% of the time. So I am not dissatisfied with that.*”	It’s important to be heard by health care professionals, and to be trusted that I as a family member know what the ill person needs	When I reach out to the health care professionals, they listen to me and I feel that they take me seriously, which makes me feel that we have a trusting relationship	Relationality	To be in the hands of the right persons
*“He had to check his blood sugar several times a day to put my mind at ease.”*	The family member has anxiety over the ill person developing more symptoms that needs handling and therefore makes him control his blood sugar	I am worried that he will get worse and that I can’t handle it if he does, I need to take control over his statues to reduce my anxiety	Corporality	The body as a tool

Initially the two first interviews were analysed together with the last author (JK, male) to ensure rigour before proceeding with the analysis. The first step of analysis is the *wholistic reading* step, which means reading each interview transcript to get a sense of the overall importance and meaning. The first author (SK, female) holistically read all the interviews, which revealed that the existentials where present in recounted lived experiences, and wrote a summarizing conclusion at the end of each, trying to capture the essence of the interview, and identifying one interview statement connected to each existentials. Relating participants' lived experiences to the existentials provided a means of understanding them. All summaries were then shared with last author (JK) who provided input. After the holistic reading the texts were read in detail (SK) focusing on identifying all statements about the phenomenon in question, the participant’s lived experiences of health care. The second step, involving *selective reading*, focused on statements or phrases that seemed particularly revealing about the phenomenon. The identified statements were read to interpret how they were expressed through and connected to the existentials. After linking each statement to one or more existentials, all statements connecting to the same existential were gathered. Some meaning units resonated across more than one theme, reflecting the interconnected nature of lived experience.

The third analytical step involved *detailed reading* where the researcher reads the statements line by line while asking the question “What does this statement/experience, in this particular existential, mean?”. Finally, the collection of statements under each existential was read as a whole to capture the collective essence. The first (SK) and the last author (JK) then described the essence of each existential through themes and thereafter captured the themes’ mutuality in an overarching theme, which finally was discussed with the remaining author (MM, female).

### Preunderstanding

The first author (SK) has many years of experience in clinical work as a registered nurse (RN) specialized in surgical care in a surgical hospital unit for oesophageal and gastric cancer. The last author (JK) has clinical experience as an RN in emergency and geriatric care and research experience related to health care and proactive, integrated care. The associate supervisor (MM) has clinical experience as an RN specialized in surgical care and in working with oesophageal and gastric cancer, and research experience with cancer processes, rehabilitation and PC. There was a collective pre-understanding that family members of those with incurable oesophageal or gastric cancer might sometimes have unsatisfactory experiences with health care, which was based on previous research and clinical experiences.

## Result

Family members experiences with health care were captured in the main theme *“To strive and struggle”.* The experiences were mainly related to the help and support directed towards the ill person and the findings clearly highlighted the strives and struggles coinciding with these experiences. Although health care experiences could be positive and satisfactory, one or more strives and struggles usually occurred. These were often found in contemplating, planning and interacting with health care. For instance, family members might experience struggles in knowing whom to contact regarding health care related need of the ill person, and which care service or hospital department to go to. This could be related both to the health care organization and to the disease trajectory. Several family members expressed that there was a continuous strive to reach the right person, at the right time and place. On the other hand, once these struggles were overcome, interacting with health care staff was often described as satisfactory. Also, family members sometimes struggled with feeling listened to by health care professionals or with being trusted for having specialist knowledge about the ill person. They expressed a general frustration and fear that the ill person would not, as they perceived it, have their needs met. This perception was often connected to the different difficulties they encountered with health care and the perception that they as family members were not being listened to sufficiently. They commonly felt responsible for navigating through and overcoming their own strives and struggles so that the ill person would have their needs met.

**Fig. 1:**
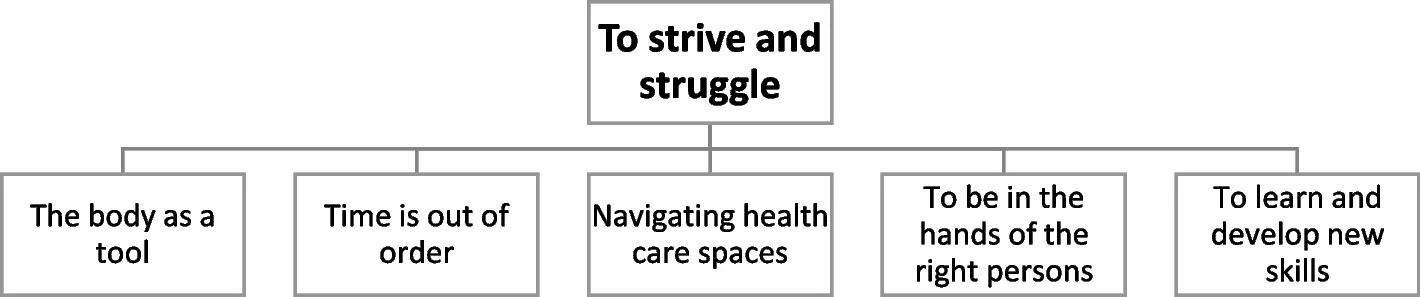
Identified theme and sub themes

The strives and struggles could be found in different areas related to the health care experiences. Five sub themes were identified describing the different areas (Fig. 1).

### The body as a tool

Family members had anxiety, sleep disorders and mental health disruptions and commonly experienced physical exhaustion particularly later in the disease trajectory. These symptoms were related to the body being seen or used as a tool and interpreted according to Van Manen’s existential of *Corporality*.

For many family members their bodily abilities were an important component in the care of the ill person. They contributed with their bodies in various ways such as assisting with bathroom visits, moving the ill person or helping them change positions and waking up in the middle of the night to manage tube feeding. This put pressure on their own physical capabilities and sometimes led to experiences of inadequacy due to physical inability to meet the ill person’s needs. In the early phases of the disease trajectory the family members often hoped to provide physical assistances whenever needed. They were frustrated and disappointed if their energy ran out. Most of the family members reported that the health care personnel did not ask them about their stamina or physical symptoms; and none of the family members thought of seeking support or asking for help. While disappointed in their lack of energy and their own inability to keep up physically with the caring tasks, they also described disappointment that their own need was overlooked by health care.

*”…being left on my own isn’t the worst thing. No, the worst thing is if he ends up bedbound and needs help with everything. Since he has a hard time walking and standing, he has to pee in a bottle. I think taking him to the bathroom is hard work.”* (No. 1)

Family members perceived that there was an expectation from healthcare staff that family members should take part in the ill person’s care, which included everything from actual physical labour to dressing wounds and handling medications. Most commonly they described being part of everyday care interventions and thus replacing home care services.

*“But she got a little help from me, she said ‘Can you take care of him’. I think that let me pitch in, at least. So he could..., no one wants to stay in the hospital, after all. So I’m pleased.” *(No. 2)

Anxiety was common among the family members and could manifest bodily. It was often related to the ill person’s symptoms or the management of those in the household. However, the interviwees described that they could cope with their anxiety up to a certain level. There was an unconscious limit within them for when their anxiety became unmanageable, and reaching or crossing that limit commonly led to family members contacting healthcare for support in managing the ill person’s symptom that was causing the anxiety. Consequently the family members monitored the ill person’s bodily symptoms constantly as well as their own anxiety related to these. When contacting health care they primarily utilized functions that were already known to them such as the contact nurses in outpatient clinics. However, if this failed emergency services or whoever was available were sought out. Just being in contact with health care personnel was sometimes a relief.

*“But actually I called the ambulance anyway, so they came and checked up on him. And took him with them.” *(No. 1)

The above was expressed by a family member who did not cope with the anxiety related to the ill person’s health status, and who called the ambulance because of lack of appropriate health care contacts.

### Time is out of order

Time was described as being totally out of order, meaning that time was not perceived as before the disease. These perspectives of time were related to the existential of *Temporality*. The family described various timelines related to symptoms, needs and health care but also everyday life. The timelines were both parallel and integrated; time progressed at a different speed for each. For instance, there might be one timeline for managing weight loss but another, usually much shorter, for relieving pain. It was evident that family members and the health care system’s time perceptions were often out of sync. Family members could for instance experience time pressure when there was a rapid decline in their ill person’s health and symptoms, while the health care would consider it normal under the circumstances. The time was also filled with uncertainty that could not be planned for or controlled. This uncertainty could be related to perceiving that pain medicine was successful today but might not be tomorrow. There was also uncertainty in not knowing when time for social interaction would be appropriate, whether work or leisure time activities were possible and how much time they would have for them. The unknown amount of remaining time before death contributed to these factors.

Time was perceived as an indicator for both good and bad health care. When health care was swift to respond with interventions and there was no waiting period it was perceived as high quality. On the other hand, when there were long waiting times this was perceived as a sign of inadequate health care. To be put on a waiting list could be interpreted as a rejection and waiting for health care could even damage the relationship with health care personnel for a long time.

*”When they were finally allowed to come in… Yes, but I do think it was very slow. Yes, but then they’ll probably say it was in the middle of the holidays. I don’t know, because I guess it was. It was in the middle of the holidays that he got ill. I’d have wanted it to be quicker.”* (No. 12)

Time being out of order was a perception that time moved quickly or slowly depending on the needs of the ill person and how that need affected their everyday life. Time moving at different speeds could sometimes be good and sometimes bad. For instance, when family members were unable to cope with anxiety, they perceived that the speed of time increased. Time being out of order and not appearing as before was in this way intertwined with the body as a tool. When the ill person had a need that was growing, increasingly becoming more unmanageable, it could sometimes also change the perception of time, as well as increasing the experience of anxiety related to that need. A well-managed need in the ill person, such as satisfying nutrition or managing pain could, lead to the perception that time was slowing down and. Seen in this sense, time moving slowly was not considered bad. In fact, time moving more slowly because needs were well managed would for some mean prolonged time until death. On the other hand, nutritional issues or unmanaged pain could lead to the perception that time was moving really quickly, which was considered a negative consequence. But issues such as unhealing wounds would only make time move slightly faster. The different needs seemed to have different effects on time and would simultaneously make it move at different rates. Time being out of order in this way could sometimes lead to family members believing that health care would have difficulties understanding their relation to time and the ill person’s health care needs, and knowing at what time they would seek help.

### Navigating health care spaces

Family members redefined the meaning of space in accordance with the ill person’s situation and adjusted their own spatiality to make room for the ill person and their needs. This concerned both formal physical spaces and personal spaces and was interpreted in accordance with the existential of *Spatiality*. Navigating through different health care spaces in order to gain access to or enter them was difficult and often considered a struggle. There was a wide perception among family members that health care staff considered formal physical spaces such as “the right” waiting rooms and “the right” treatment place more important than the ill person’s or the family member’s need at a given time. It was, however, not always clear which spaces were meant for what purpose and in which spaces family members were welcome.

*”’I’m in so much pain that I don’t that I don’t know what to do. ‘And it didn’t matter what he did. No, but when we tried to call it in, it was… yes, it was two minutes past four, I guess, no one was answering in the oncology department, or in the surgical department, or anywhere. I thought we’d have to drive to the emergency department then. So we drove to Hässleholm. ‘No, we don’t accept anyone here. This is surgery. You have to go to Kristianstad.’”* (No. 4)

*”Then it was… we called the radiology department, and they referred us to this [inaudible 0:12:57] a bit like this, that was probably the surgeon, or… so, we didn’t know how it worked, between the departments. So it was very difficult to get information like that. And we left… we left then for the emergency department.”* (No. 6)

Finding the right place to get help for a specific symptom or need could be challenging and stressful. This added to the struggle and often led to frustration among family members. On the other hand, the right spaces were often related to what was perceived as good health care. That is, being in the right place for the right need was crucial for the perception of the care received. Trying to navigate through the different spaces in the health care system or not being in the right place created a new space, the in-between space. This was a space the family members commonly found themselves in, which could lead to confusion, anxiety and frustration. The in-between space consequently added to the already strenuous situation and resonated as anxiety in the family members’ bodily experiences.

As mentioned above, family members adjusted their own space to make room for the ill person’s spatiality. Their personal space consequently fluctuated as it was deemed less important than the ill person’s. As a result, a personal in-between space was sometimes created. This was expressed as less stressful than the in-between space created by formal physical settings.

Emergency departments were often considered by family members to be somewhat of a struggle and not an appropriate space for the ill person. The emergency environment could be perceived as messy, and unsuitable for frail ill persons; and lacking in knowledge of the specific disease and needs. It could cause family members to feel worried, upset or disappointed. Several family members expressed the wish for an emergency place where specific palliative and cancer care knowledge could be found in an environment that was responsive to the state and needs of an ill frail person and their family members.

### To be in the hands of the right persons

It was highly visible that relationality was of great importance when it came to health care experiences. The strive to establish good relationships with health care staff was part of the struggle and became important for most of the family members interviewed. The sub-theme therefore drew on the existential of *Relationality*. A good relationship by and large meant to be seen and/or be listened to. In their pursuit to be seen and heard they navigated through various channels and structures, like contact nurses or home care services, with varied results. Being successful in this navigation often meant meeting the right person at the right time, in the right place. However, a lot of the times being successful in meeting the right person was down to luck. The right person was generally described as health care personnel with a good will, and with the intent to do good regardless of the situation, and to display humanity or go the extra mile for the ill person. This would almost always mark a good or trusting relationship, albeit brief, sometimes even more so than having the perceived right knowledge of the disease and situation and would result in a reduction of family member’s struggles.

*“Yes, let me tell you that I was with him once before, and then I didn’t like her. But it was the same doctor? Yes. She was really good now. And then I thought, when… yes, then she was really human.” *(No. 9)

*“That you can call them. I noticed yesterday, he was so happy when the doctor called. You feel worthwhile then. Like you’re not forgotten. You know, then the disease is less significant than the person. So we have absolutely nothing…, on the contrary. The contact. To be able to speak from your heart.” *(No. 2)

Having a good relationship with health care staff was also intertwined with time and space. Not getting help when needing or not finding the right place could shape a relational experience. For example, when health care professionals took a long time to get a hold of or when the space associated with a professional was confusing or unreachable, the relationship could be affected in a negative manner. This meant that even before establishing relations a meeting could be deemed inadequate or unfulfilling by the family member, and this would negatively affect the interaction. On the other hand, finding help at the right time and in an accessible space could positively affect the interaction with staff, making the relational experience good, regardless of the manner in which the health care personnel met them. Moreover, a professional with the ability to see, listen and act upon the needs presented could in any case turn negative time, space and interactions around creating a positive relationship for the family member going forward.

For some, a good relationship meant having their need for control met. One way of this happening was getting sufficient information (sufficient according to the perception of the family members), regardless of the information needs of the ill person. This way of staff of enabling control felt equal to a trusting and well-functioning relationship. Staff not enabling control, on the other hand, could lead to experiences of inadequate health care relations and increase the family member’s struggles.

### To learn and develop new skills

New materials were continuously introduced to family members as health care experiences in the ill person’s trajectory, such as pain patches and syringes. Handling new materials meant learning new skills and increasing the individual family member’s health care knowledge, which were lived experiences interpreted according to Van Manen’s existential of *Materiality*. Learning and developing new health care skills was something family members often wanted to do although it was often perceived as something they were compelled to do: they sometimes experienced an underlying expectation from health care professionals to learn and develop new skills, and to care for the ill person at home using these skills as a token of an engaged and caring family member. Most family members would nevertheless partake in the process of learning new skills. Initially experiences with new materials such as feeding tubes or wound dressings were somewhat unfamiliar and sometimes even scary for the family member. As time progressed such obstacles were usually overcome, and the majority were glad to aquire new skills while at the same time being proud to have the ability to master the situation. Most new skills were eventually experienced as a natural and integrated part of everyday life together with the ill person.

*“You think it’s awful that you get hit with this… that someone in your family gets hit with this, and then it’s like ‘Yes, but I can show you this,’ he says, ‘How you should… ‘ ‘Yeah, I’m not doing that,’ I said, ‘I’m not going near those tubes and the thing on their stomach,’ I said. ‘I’m not doing that.’” *(No. 8)

However, a small group described that they were not willing to learn and develop new skills. They would communicate this to health care staff. They made a point to solely be a spouse and family member and not a carer or a resource, thus refusing to oblige to the perceived underlying expectation. Their motivation was mainly that they did not want to alter the relationship with the ill person; alternatively, their refusal might be a consequence of an already perceived tainted relationship with health care professionals. Therefore, the refusal was not necessarily directly related to the new materials or skills. Finally, some family members would negotiate with health care staff about not participating in home care because they did not feel the need to do so in order to feel engaged with and integrated in the ill persons situation.

## Discussion

The findings showed that several dimensions of family members’ health care experiences were perceived as a struggle. One was family members’ anxiety and how it was related to the ill person’s symptoms. Anxiety was often an indicator and a deciding factor for when to seek healthcare for the ill person, and even sometimes a more significant assessment of the situation for family members than the severity of the symptom. It is a well-known fact that being a family member of someone who has cancer can cause high levels of anxiety. Previous studies suggest that it is important for health care to pay attention to psychological challenges of family members, such as anxiety, and to consider which factors might indicate the need for support [45–47]. There are several ways of providing anxiety support, including psychoeducational interventions or providing coping strategies for psychological balance. However, the effects are often unclear [9, 48–53]. A concrete way of reducing anxiety and stress for family members of those who are incurably ill, with reported positive effects, is palliative care-planning. This is a structure used by staff, and it involves the ill person and their family members co-creating a plan based on care-related needs and preferences throughout the disease trajectory [54, 55]. Furthermore, research shows that family members who are or learn to be emotionally attentive increase their quality of life when they focus on their own emotions. However, when their emotional awareness focuses on others, the risk of negative effects in their daily lives increases [56]. It should be noted that not all family members may need interventions targeting their mental health and, in some studies, only those with pre-reported distress benefited from such [57, 58]. Even so, the findings of this study suggest that addressing family members’ anxiety is an important factor to consider in PC and providing support for it may contribute to less anxiety connected to the ill person’s symptom management in the home.

The findings also highlight that when the family members decided to reach out to health care, they often experienced the navigation of the different healthcare spaces as a struggle and understanding the formal health care spaces was often a cause of frustration. As a result, family members sometimes saw no alternative but to seek care through emergency departments, even though they considered them to be an inappropriate environment for both the ill person and for themselves. According to previous research, these experiences of health care are not unique to the family members of this patient group but have also been observed among families of other individuals with advanced cancer [19, 28, 59]. The present results, however, show the navigation struggles in relation to the specific symptoms of this fast progressing cancer and contribute to the understanding of this patient group’s family members’ specific need for support from the health care system. Health care systems in general seem to lack the ability to meet the needs of the incurably ill when expressed by their family members or informal carers, and often lack systems for involving them, which contributes to the challenges of navigating health care. Research shows that providing information about healthcare organization and the different departments’ roles in the disease trajectory can relive some of the struggles connected to navigation and contact challenges experienced by family members [19, 28, 29, 59]. Such information and support can be part of integrated cancer care [60]. Integrated care is the alignment of health and healthcare services to meet the needs of the individuals. It is considered the opposite of fragmented care, which can be the cause of struggles in navigating struggles of health care systems [61–63]. The navigation struggles in this study indicate the need for PC to be integrated and for family members to be included in that integration. Integrated PC is recommended by the American Society of Clinical Oncology [5] and studies show that early PC that is integrated has positive effects for family members concerning information and guidance through the disease course [24, 25]. One way of providing early PC in an integrated manner that leads to guidance is through outpatient clinics [5]. The evidence for the efficacy of care by palliative outpatient clinics is extensive and shows that emergency and hospital utilization can be reduced and end-of-life experiences improved [64–66]. However, an outpatient clinic in itself is not necessarily functional unless the services provided are useful for the patients and their family members. Our findings indicate that useful structures are those targeted to integration and services that relieve the struggles of navigating of the health care system. One such service is presented by the Swedish National Cancer Strategy [67] which suggests that informal caregivers should have the possibility to contact health care for palliative cancer care consultation all hours of every day. Based on the findings and prior research, early enlistment to a palliative outpatient clinic which provides support to family members in the form of increased integration with cancer care guidance is suggested and could be provided through concrete arrangements such as around the clock accessibility through giving out 24/7 contact numbers and providing emergency consultations.

While navigating the health care system family members often find it difficult to establish good and trusting relationships with health care personnel; in the present study, luck was considered a significant factor for such relationships to form. However, an additional factor may also be that some family members were too busy navigating the system or worrying about it to make actual room for such relationships when available. Alternatively they had already formed trusting relationships with specific contact nurses and therefore did not experience the need for new relationships based on luck during unexpected events, for instance. In the description of the theoretical framework underpinning “Fundamentals of Care” [68, 69] the relationship with health care personnel is seen as the foundation of care and the trusting relationship is central to experiences of good care, without which such care cannot be achieved. The theory behind “Fundamentals of Care” describes how establishing trusting relationships is sometimes more important than having a specific need met. In this study these ethics could also be identified. Bearing this in mind, focusing on establishing trusting relationships for family members of those who are incurably ill ought to be a priority of health care, which needs to be organized to enable such relationships [70–72]. The results of this study imply that the difficulties navigating the health care system left no room for establishing trusting relationships. Other research has shown that health care personnel may also lack the ability to form trusting relationships with the incurably ill and their family members. This inability, then, presents a hindrance for the important existential conversations to take place within PC [73]. Although establishing a trusting relationship with family members can arguably have a positive effect on the health care experience, early integrated PC would possibly have the same result. Since PC is based on the needs of both the patient and the family members, meeting those needs ought to include establishing the preferred relationship of the care recipient [18, 20, 74]. However, perhaps fundamentals of care practised through early integrated PC is the preferred approach. The findings suggest that establishing a trusting relationship early and creating opportunities for health care personnel to connect the palliative approach with fundamentals of care may contribute to reducing the role of luck as a key factor for crucial relationships in cancer care.

### Method discussion

In qualitative design, trustworthiness is assessed by evaluating credibility, transferability, confirmability and dependability [75, 76]. Credibility, which relates to the believability of the results and significant representation of the experiences of the participants, was ensured by capturing of variations in experiences related to the disease trajectory, predominantly lengthy interviews which continued until no new data emerged, open-ended questions to ensure that the data was rich, co-constructed notes on the visible timeline during each interview, author triangulation and presented quotations. Transferability, the possibility for a study to be transferred to another setting or participant group, is judged by the reader. In this paper, a clear description of the setting, participants, data collection and analysis process is given for the reader to assess transferability. Considering sample and data, the results may be transferable to other groups of family members of persons with similar advanced diseases. Confirmability, how well the results are grounded in the data and methodological factors underlying this, was increased by the use of triangulation, the consideration of the authors’ pre-understandings throughout the process, the description and alignment of Van Manen’s analytical steps, and presentation of data grounded quotations. Dependability, that is rigour in the process and consistency in the findings over time, was enhanced through a consistent semi-structured interview format, including the same open-ended opening question and flexibility to explore diverse experiences. The visible timeline utilized in all interviews and the co-authors involvement and the arising discussions leading during the analysis process also increased dependability.

### Limitations

Although measures of trustworthiness were taken, study limitations occurred. A limitation related to dependability was the fact that the age of only half of the participants was recorded because question was missing in the interview guide. Other weaknesses had to do with recruitment shortages concerning reminders and follow-ups, which possibly contributed to the large non-response rate.

Patients defined who constituted their family members, and those individuals were eligible for inclusion in the study. Despite this selection process, all participating family members were female, with partners representing 83% of the sample. This may be explained by the fact that 52 of the 68 patients who were initially contacted to identify family members were men and that all 12 family members who participated were close relatives of a male patient. Nevertheless, the findings mainly reflected the perspectives of family members who were female partners sharing everyday life with the ill person. Other possible family members such as parents or siblings were scarcely represented in the sample. This may have led to the result being influenced by a more traditional gender role. However, the sample reflected the main demography of the group in a relevant manner, in which men constitute the majority of diagnosed cases (70,4% Oesophageal cancer, 61% Gastric cancer) [77].

## Conclusion

This study adds to the knowledge of health care experiences of family members of those with incurable oesophageal or gastric cancer. It suggests that supporting and guiding family members through the fragmented health care system by introducing integrated early PC may decrease anxiety, increase the likelihood to form trusting relationships and lead to fewer emergency department visits.

## Supplementary Information

Supplementary Material 1.

## Data Availability

Analysed data are available from the corresponding author on reasonable request.

## Abbreviation

PC: Palliative Care
