# Supplementary material for: Experiences of health care in family members of persons with incurable oesophageal or gastric cancer – a hermeneutical phenomenological study
Source: BMC Palliat Care. 2026 Oct 13;25:301. doi: 10.1186/s12904-026-02380-x (PMC13663004; doi:10.1186/s12904-026-02380-x)
Supplement: Supplementary file 1 [file 12904_2026_2380_MOESM1_ESM.pdf]

Interview Guide

Information

I am a nurse and a doctoral student at the Palliative Care Development Centre and Lund University. The research project I'm working on is a collaboration between the surgical clinic and Lund University. The purpose of this conversation is to learn more about your experiences with the care that you and your loved one have received.

I will be asking questions, and of course, you decide what and how much you wish to share. We've set aside approximately 1 to 1.5 hours, but it may vary, and you are free to let me know if you wish to stop at any time.

The interview will be recorded and later transcribed into text. The text will be analyzed and processed, and in the final results, no one will be able to identify you.

Some questions may seem a bit unusual, but they are often intended to help me gain a deeper understanding of what you're sharing. If we stray too far off topic, I'll gently steer us back to the purpose—mainly out of respect for your time.

On this paper, you'll see a timeline. Based on that, you can describe how you and your loved one have experienced contact with the healthcare system. **Tell me, how did the journey begin?**

Follow-up Questions:

- When you say… what do you mean by that?
- Can you tell me more about that?
- How did you experience it?

Timeline Interview nr _______________
